# Increasing the Potential Effectiveness of Universal School Meals through Community- Engaged Implementation Mapping: A Qualitative Case Study

**DOI:** 10.21203/rs.3.rs-8902980/v1

**Published:** 2026-04-19

**Authors:** Angel Smith, Divya Kulkarni, Sofia Dubrin-Meneses, Resa M. Jones, Jonathan Deutsch, Jacob Kurtz, Irene Hong, Hillary Kane, Senbagam Virudachalam, Shiriki Kumanyika, Ross Brownson, Gabriella M. McLoughlin

**Affiliations:** Barnett College of Public Health, Temple University; Barnett College of Public Health, Temple University; Barnett College of Public Health, Temple University; Barnett College of Public Health, Temple University; Drexel University; Reinvestment Fund; Feeding Philly; University of Pennsylvania; Children’s Hospital of Philadelphia; University of Pennsylvania; Washington University in St. Louis; Barnett College of Public Health, Temple University

**Keywords:** School Meals, Implementation Mapping, Community Engagement, Health Equity, Policy, Food Insecurity, Nutrition, Childhood Obesity, Getting to Equity, Exploration Preparation Implementation Sustainment

## Abstract

**Introduction:**

Universal School Meals (USM) serve as a first line of defense for students facing hunger in marginalized communities. Despite this, school meal uptake remains low across the district due to several barriers in the inner and outer school setting. Community- and school-led interventions and strategies are an underutilized approach for enhancing USM implementation and student uptake but may yield a more sustainable impact on addressing food insecurity. The aims of this study are to 1) engage in a structured implementation mapping process with a diverse array of school and district representatives grounded in implementation science and health equity frameworks, and 2) understand the key constraints and supports of implementation mapping from the lens of policy implementers and recipients.

**Methods:**

We recruited 4 schools (2 intervention, 2 waitlist) to engage in the mapping process. Meetings covered tasks 1–4: steps 1) needs assessment, 2) identify target objectives, 3) select strategies for enhancement, and 4) prepare an implementation plan over 4–5 planning meetings in partnership with school food service, students, parents, and teachers, and administrators of each school. Transcripts were deductively coded using the Exploration, Preparation, Implementation and Sustainment (EPIS) and Getting to Equity (GTE) frameworks and inductively by the research team.

**Results:**

We engaged 19 participants across the 2 intervention schools (3 teachers, 2 food service, 2 parents, 2 administrators, 10 students). Data from the mapping meetings were primarily coded to *Culture* from the EPIS framework and *Increase Healthy Options* from the GTE framework. Code relations analysis between both frameworks highlighted factors such as school and district operations and social drivers of health among school communities impacting equitable USM implementation. Thematic coding yielded four primary themes: Infrastructure/Material Barriers, Communication, Stigma & Social Drivers, and Autonomy & Decision Making. These themes highlight the facilitators and barriers to implementation, mapping, and changing school meals on a systems level.

**Conclusion:**

Engaging school and district partners is an essential step for building lasting changes in the health of underrepresented communities. We successfully engaged a group of representatives to facilitate implementation mapping and implementation strategy development. This is one example of how community-partnered research can be leveraged to potentially facilitate change in school systems.

**Registration:**

ClinicalTrials.gov, NCT06579079, Registered on 07082024.

## Introduction

Access to nutritious foods in childhood is essential to eating a balanced diet and preventing chronic disease in adulthood. Unfortunately, food insecurity (the lack of consistent access to food) has risen in the United States over the last several years and particularly affects marginalized communities such as Black and Hispanic populations and those living in low-income households making them more susceptible to poor diet quality and development of chronic diseases (1–4). Universal School Meals (USM) is a federal program linked to the Community Eligibility Provision of the National School Lunch Program that offers myriad benefits for students and families, given their impact on reducing food insecurity, improving diet quality, and promoting child health (5, 6). USM is beneficial for providing students with two nutritious meals per school day and potentially reducing the overall household expenses for food. Despite this, student participation in USM in Philadelphia remains low (7). Several factors, such as stigma associated with free meals and organizational challenges affecting school districts, limit the implementation and effective reach of the USM policy. Innovative implementation strategies (i.e., implementation interventions) can be useful in bolstering the delivery of USM and optimizing student benefits as a result.

Implementation science (IS) methodology is designed to ensure that evidence-based interventions achieve their intended outcomes for their target population and address program/policy interventions at all levels (i.e., adoption, implementation, and sustainment) through pragmatic changes in the environment (8, 9). Furthermore, policy effectiveness may be enhanced by integrating lessons from the experiences of program stewards and recipients, refining plans, and measuring fidelity (10, 11) IS utilizes implementation mapping (IM) - an adapted version of intervention mapping - to facilitate co-development of implementation strategies to support translation from research to practice (7, 12–15). IM comprises five tasks, including a needs assessment, determining outcomes and performance objectives, selecting strategies, developing protocols, and strategy evaluation. This method has been used in prior research for translating evidence-based research into practice and enhancing the reach or effectiveness of an existing intervention (12, 14). A study by Byhoff et al. utilized implementation mapping to increase the reach of SNAP participation through the development of multi-level implementation strategies that improved community enrollment and administrative processing (16). Specifically, they used collaborative planning via weekly work group meetings with community and academic partners to ensure community voices were amplified in the mapping process. This selection placed decision-making power in the hands of community experts and policy/program recipients; however, it excluded the perspective of youth recipients.

Implementation science seeks to ensure that evidence-based interventions achieve their intended outcomes for their target population and address program/policy interventions at all levels (i.e., adoption, implementation, and sustainment) through pragmatic changes in the environment (8, 9). Furthermore, policy effectiveness may be enhanced by integrating lessons from the experiences of program stewards and recipients, refining plans, and measuring fidelity (10, 11). Examples of implementation mapping often lack the perspectives of implementers (e.g., school staff, district staff) and recipients (e.g. students, families) as key drivers of the process. For instance, one study to improve peripartum pain management through implementation mapping engaged several implementers from the healthcare center yet did not include patients (17). Overall, this process was driven by clinical implementers of the program; however, it did not engage the perspectives of birthing people during the development stage (17). Other research that aimed to build organizational readiness to enhance sexual assault prevention was successful in creating a multi-site intervention driven by organization members stretching across several levels of intervention (14). However, this work excluded the perspectives of previous program recipients. Accordingly, there is a need for better inclusion of underrepresented voices in research and community engagement to drive equitable policy implementation.

In this article, we report results of a detailed case study of a community-partnered implementation mapping approach to co-creating USM implementation strategies in Philadelphia, PA. The aims of this study were to:

1. Engage in a structured implementation mapping process with a diverse array of school and district representatives grounded in IS and health equity frameworks.
2. Understand the key constraints and supports of implementation mapping from the lens of policy implementers and recipients.

Our comprehensive approach included typical school partners (i.e. administration and teachers), as well as program providers (i.e., food service staff) and recipients (i.e., parents & students) groups that are often excluded from key implementation decisions (18, 19). This involvement is designed to empower community members’ voices that have been excluded from or overlooked in past policy implementation processes.

## Methods

The School District of Philadelphia (SDP) is a large district with 331 schools and a total enrollment of 198,299 students (20). The principal investigator, GMM, has been providing ongoing (unpaid) support for the evaluation of school nutrition programs and the development of school nutrition policies. This IM project builds upon our previously published needs assessment (IM Task 1) that identified main determinants of school meal uptake in the SDP, including strong leadership and social/organizational barriers (21). We described initiatives to address these determinants using implementation mapping tasks 2–4 to co-create implementation strategies to enhance the delivery of USM. The analysis is grounded in IS and equity-based frameworks: Getting to Equity (GTE) and Exploration, Preparation, Implementation, and Sustainment (EPIS) to best understand the key constraints and supports of implementation mapping through the lens of our stakeholders. GTE is an equity-oriented framework for implementing obesity prevention policies and programs, including those related to equity in food security and diet quality. This framework guides thinking and planning to center health equity in policy, systems, and environmental (PSE) work (22,23). The EPIS framework describes all phases of implementation and the interactions between outer systems of influence and internal organizational structures (24). Using these frameworks allows for a better understanding of the spheres of influence within USM implementation and mapping strategies to be most suitable for USM recipients and implementers. This paper does not report outcomes of the clinical trial itself since it is still underway, but an *in-depth qualitative case study* of how implementation mapping processes take place within the context of an ongoing clinical trial.

### Recruitment of IM Participants

The broader aspects of the study setup and randomization were previously published in our type III hybrid trial protocol paper (12). Briefly, four schools were selected for the trial, then organized into intervention and waitlist groups using cluster randomization. Schools were matched based on demographics, size, participation rates, and household food security levels.

This case study describes the process for IM tasks 2, 3, and 4 with the first-round intervention schools, i.e., as opposed to the schools on the waiting list. As noted previously, IM task one—the needs assessment had already been conducted. At the time of this writing, the research team is executing task 5—strategy implementation and adaptation. To maintain confidentiality, all school strategies from the supplemental materials and findings from task 5 will be reported subsequently. The research was approved by Temple University Institutional Review Board (IRB) #28959; all participants provided written informed consent and received $25 in compensation for participating in each meeting.

Mapping intervention tasks 2, 3, and 4 were to involve a series of meetings with representatives of each of the two schools. The schools were responsible for recruiting meeting participants including students, teachers, parents, staff (climate, community coordinators), and administration (principals, counselors, case managers). To aid in recruitment, the research team developed a flyer for school leaders to disseminate that included QR codes to a website link and information about what each meeting entailed (Supplement A). In some cases, the research team assisted in recruitment by speaking directly to potential participants. At the first meetings, participants provided written consent and/or assent. All participants received a $25 gift card for each meeting they attended.

#### Intervention:

##### Collaborative Implementation Mapping

Participating sites included two K-8 schools with satellite kitchen service (i.e., schools serving meals prepared off-site). Each school recruited a collaboration group comprised of 7–9members of the school community. Across both schools, meeting groups consisted of students, parents, teachers, administration, and food service workers. Each member was present for at least one task in the IM planning process.

#### Meetings

We held nine meetings (4–5 meetings at each school and respective recipients) to address IM tasks 2–4, as shown in Figure 1. We developed three implementation mapping workbooks (Supplement B), one for each of the three relevant tasks, that in combination, detailed the mapping steps to develop implementation strategies to enhance impact and uptake of USM (21). The research team’s community advisory board (CAB) reviewed the materials to ensure suitability for our audiences.

At the time this paper is published, the research team will be executing task 5 (September 2025-May 2027)—strategy implementation and adaptation. To maintain confidentiality, all school strategies and findings from task 5 will be reported subsequently.

##### Meeting 1: Task 2—Determining Outcomes

The research team presented the needs assessment findings from Task 1 to help participants to understand the main school meal implementation barriers and facilitators within their respective school (21). School teams were informed of goals of the project and prior research activities and results from IM task 1 during the meeting. The Task 2 workbook included an infographic of our needs assessment findings alongside individual and group reflection questions such as “What do students, staff, and parents think about meals in your school?” and “What are some challenges or concerns around school meals in your school?” (Supplement B). These questions elicited broader discussion to guide the research team toward priority areas for meeting 2.

##### Meeting 2: Task 3—Selecting Strategies

Meeting 2 guided participants to state the main achievable objectives and create/select strategies based on a list of suggestions built upon the School Implementation Strategies, translating ERIC Resources (SISTER) and Implementation Strategies Applied in Communities (ISAC) strategy lists that were compiled in our implementation mapping manual (Supplement C) (25,26). The workbook included guiding questions to allow the school community to reflect on their needs and assets (Supplement B). From this, the school groups developed specific strategies aligning with various categories based on the outcomes identified in Task 2. Once the strategy list was introduced, we used several discussion questions to guide the selection and development of a strategy list, such as “Do you see anything from this list happening in your school already?” and “From this list, what do you think is needed in your school setting?” Once the strategies were chosen, the groups ranked them as easy, medium, or hard, and identified any barriers to implementation based on their perceptions of feasibility. This step allowed the group to brainstorm realistic implementation steps. Finally, we organized strategies into categories for the inner and outer settings, and short-term or long-term changes.

##### Meetings 3 & 4 or 5: Creating A Plan

The final task guided schools through creating an implementation plan. We provided a final workbook to outline what implementation looks like and how to achieve the desired outcomes set in Task 2 (Supplement B). For this, we identified strategy leaders, outlined action steps for effective implementation, and determined all necessary resources and support. Participants were given prompts, including “What do we need to implement these strategies?”, and “Outline the name and title of leaders for each strategy then assign support” to guide planning.

##### Data Analysis: Aim 1

All meetings were recorded and professionally transcribed and analyzed using MAXQDA qualitative analysis software (27). Transcripts were deductively coded in four steps: 1) generating codes for the EPIS constructs, 2) applying data codes to the phases of EPIS, 3) aligning codes to the GTE, and 4) examining alignment of the framework coding through code relations analysis. Coding transcript segments into buckets allows us to understand the significance of various constructs when building strategies.

Before coding, the research team reviewed all original EPIS domains and constructs. EPIS definitions were previously applied to implementation in healthcare settings; therefore, our definitions were tailored to fit school-based nutrition interventions based on our IM data analysis protocol (Supplement D). Each construct name and definition was modified for use in school-based qualitative data analysis. Constructs not aligning with the school community were excluded or revised to fit the context. For example, *Patient/Client Advocacy* was previously defined as “support or marketing for system change based on consumer needs, priorities and/or demographics.” The title of this construct was renamed to “*Responsiveness to School Community*” and the definition was tailored to, “leadership response to school needs and priorities in the school environment/operations.” Construct names and definitions were changed for both EPIS and GTE based on consensus from the research team. The coding scheme was reviewed by the CAB during bimonthly meetings to discuss the relevancy of coding constructs and clarity of definitions.

Phase one included coding to one or several constructs and domains. Specifically, we included eleven constructs from EPIS, representing four domains. Domains were defined as follows:

##### Step 2: Applying Data and Codes to Specific Phases of EPIS

The four phases of EPIS outline specific steps of implementation: Exploration, Preparation, Implementation, and Sustainment. We assigned codes to EPIS based on the stage of implementation we were in. Accordingly, codes from tasks 2 & 3 primarily fit into the exploration phase, while codes from task 4 were coded to both Exploration and Preparation. Within each phase, codes were separated based on whether they were presented as barriers or facilitators to implementation as shown in Table 2.

##### Step 3: Applying Codes with the Getting to Equity (GTE) Framework

The GTE framework allows the research team to ensure that equitable implementation practices were embedded in the mapping process. Each construct touches on a crucial element necessary for equitable obesity prevention programs. The codes were divided into categories of barriers or facilitators to identify code segments that indicate a challenge or support for potential strategies. Constructs are defined as follows:

##### Step 4: Establishing coding consensus

We conducted a code relation analysis to determine the overall alignment between the frameworks. We pulled coding reports from MAXQDA to understand the overlap between GTE and EPIS constructs. Using this, we identified quotes that were coded to one construct within each framework to capture impact and relationships between constructs such as EPIS *Culture* and GTE *Reduce Deterrents*.

To ensure our approach was rigorous, we followed guidance from qualitative research experts to inform our methods of coding and consensus (28,29). The research team established consensus for each coded document, and all transcripts were coded at least 2 times. A primary coder reviewed and assigned codes in all 9 transcripts, while 2 secondary coders coded 4–5 transcripts each. To establish consensus, secondary coders met with the primary coder to review code line-by-line and resolve discrepancies.

##### Data Analysis: Aim 2

To achieve Aim 2, we conducted an open, inductive thematic coding process to identify prominent themes within the data throughout the mapping process (30,31). The inductive coding followed three major steps: (1) creation of major themes, (2) coding in MAXQDA, and (3) peer debriefing. Three coders were randomly assigned one transcript to review and identify major themes. Each coder developed a short list of themes and definitions with corresponding quotes for context, then collectively selected a final list of themes. Each coder was assigned three transcripts to code using the inductive coding schema, and coders used a shared reflections document to identify quotes for review in a coding consensus meeting. The team met weekly to review coding results for their transcripts, make changes to the coding schema, and refine coding assignments. The codes underwent two rounds of revisions: subthemes were first split up, then redefined for granularity. Once the coding schema was finalized, each coder coded three transcripts to complete coding of all data (9 total).

##### Peer debriefing

After the final round of inductive coding, the research team recruited peer debriefers and project mentors from their established CAB via email. We shared a description of the project with all members, and interested respondents were included in the process. We recruited 5 debriefers (3 community advisory board members and 2 researchers). The mentors comprised academic researchers in public health, epidemiology, and IS from several universities and research groups. CAB members included individuals from local city-affiliated organizations, non-profit groups, and the school community (i.e., parents, students, and food service).

Peer debriefers participated in completing a two-step code system review and coding reflection. Each debriefer was assigned a document to review, which they submitted with a brief reflection on their feedback. Three debriefing meetings were held with peers to review the documents. Upon completion, feedback was distributed to the group for a final review. We had participation from 4 of the 5 reviewers in the final round of peer debriefing. Feedback from our discussions was incorporated into the coding scheme, which was revised accordingly. Following the revisions, the new coding scheme and assignments were distributed to the debriefing team for a final review. Peer debriefing was used in Aim 2 to ensure the inductive approach was rigorous. Six CAB members and 2 college faculty volunteered to participate in reviewing all themes and coded constructs.

## Results

Across both sites, there was a total of 16 participants. Meeting participants comprised of teachers (n=3), food service workers (n=2), parents/caregivers (n=3), school administrators (n=4), and students (n=4). Majority of respondents identified themselves as Black/African American (n=11). Respondents were predominantly English-speaking. Age varied greatly depending on the group; however, most participants were between 30–49 (n=8).

### Exploration, Preparation, Implementation, Sustainment (EPIS)

The most common construct within EPIS Phases was Exploration - Facilitator. Among EPIS Domains, the Inner Context was commonly discussed; three constructs are categorized underneath the Inner Context; with *Culture* as the most coded construct. EPIS Outer Context also has seven subthemes including the critical topics of Service Environment - District & Vendors and Interorganizational Networks and Partnerships. Table 5 shows the distribution of coding across the EPIS Domains and Constructs coding schema.

Across both schools, coding frequencies changed over time. At school 1, *Culture* was the most coded subtheme among EPIS constructs. The importance of specifying culture waned over time with the frequency of codes to this construct decreasing in later meetings. Themes in *Culture* pertained to cultural relevance of meals, stigma, and student meal preferences. Across student, staff, and caregiver participants, several reported witnessing or experiencing stigma related to school meals.

For outer context constructs, changes to coding over time were most prominent for Service Environment—School District and Vendors. At school 1, this construct was most prominent during meetings 1–3, whereas at school 2, coding was most prominent during meetings 1 and 3. Exploration was a major theme in meetings focused on tasks 2 & 3; meetings for task 4 had more codes to Preparation. Exploration was a major theme during the initial meetings. Approaches to the exploration stage varied across school sites. School 1 focused on slight changes they could make to operations within their school to make meals more engaging for students. For example, food service emphasized the importance of giving students autonomy over the type of food they will be served by including them in decision making processes. School 2 used tasks 2–3 to 2 focus on building stronger systems of outreach and enhancing educational opportunities for their school community. They explored top-down interventions that influence the way students participate in meals. This included examining their school-level goals related to food justice and expanding resources for students and families. Changes to the community included installing a garden that aligned with their goal of creating a local farm-to-table model for their students.

### Getting to Equity (GTE)

Several constructs coded to EPIS were included in GTE, either as a barrier or facilitator (Table 5). The most coded strategy constructs were Increase Healthy Options and Community Capacity - Strategy. In school 1, the Increase Healthy Options strategy remained high among all tasks with a range of 17–20 codes. Conversely, school 2 had an increase in codes of *Increase Healthy Options - Strategy* signifying a strong desire for changes targeted towards desirability and access. Themes that arose in increase healthy options strategy were existing factors within the school community that make elements of school meals more accessible (i.e., school menu distribution, breakfast in the classroom, food sharing areas), meal preparation strategies, and programs that support healthy food consumption and/or perceptions in the school (i.e., cooking classes, food distributions). For example, a quote from school food service details innovative meal preparation that ensured students were taking and consuming breakfast:

“They used to give us bags. I used to put the hot food in bags to make sure it stayed hot. Now I put it in there and it still be nice and hot. As long as it’s nice and warm, they would eat it all. They would eat it all.” - Food Service

Among GTE barriers, *Increase Healthy Options* and *Reduce Deterrents* were the most coded. School 1 had the most codes to Increase Healthy Options strategy during the first 4 of 5 meetings, whereas school two coding had very few variations in frequency over time.

### Alignment between EPIS and GTE frameworks

In cases where more than ten coded segments were aligned with both frameworks, coded extracts were added to illustrate this alignment. In the case of twenty or more segments that are aligned, cells appear in dark green with white text. The most alignment was seen with *Increase Healthy Options - Strategy* (GTE) and *Innovation Factors* (EPIS). This demonstrates that in mapping meetings, ideas from participants often referred to improving school meals (innovation factors) through increasing the appeal or the quality of food, thus enhancing access to USM. Quotes from the meetings identified stigma and negative perceptions within the school environment that impacted meal uptake, thus signifying a need for strategies to change the social perceptions of school meals. For example, an administrator said, “We have a school Instagram account. So maybe the teachers that are part of running that could do maybe a weekly food highlight with a picture and make it cute looking”, highlighting the ways they can increase access to school food through mass media targeting students and families to improve perceptions and appeal.

There was less alignment between the GTE, and several EPIS constructs such as funding, leadership, service environments—policies, and responsiveness to the school community. These constructs had less than 10 coded segments that were included in EPIS and GTE. Further examples of code relations can be found in Supplement E. which illustrates the alignment in coding to both the EPIS and GTE frameworks by showing the frequency of coded segments that were assigned to both frameworks.

### Aim 2 - Barriers and Supports to the Implementation Mapping Process

Upon the final debrief meeting, we arrived at four primary themes and thirteen subthemes, displayed in Supplement F. After the second round of open coding, two new codes were added. Upon finalizing coding for all nine transcripts, themes were redefined and reorganized based on feedback from the research team. The most coded primary theme from our inductive coding was *Communication*. Of the four subthemes, community cohesion and marketing/awareness were the most coded. While community cohesion was an original subtheme, marketing/awareness was added by peer debriefers.

The theme of *Communication* encompasses several subthemes related to communication between schools, the district, and partner organizations that impact operations and implementations. The subthemes include *Community Cohesion, Bidirectional Learning, and Opportunities for Improvement Marketing/Awareness. Community Cohesion* highlights the degree to which there is a linkage between the schools and the community. This includes successes and/or issues in the way programs are delivered and their impact on overall effectiveness. Further, bidirectional learning is defined as an exchange of information about roles and responsibilities among meeting participants. Meetings served as a chance for colleagues to learn from each other about policies or operations that impact meal service. *Marketing/Awareness* encompasses any messaging around school meals that come from implementers. Meal promotion attempts from the school district or food service could increase participation. Lastly, *Opportunities for Improvement* was a subtheme to capture areas of frequent miscommunication that could benefit from improved channels of communication between the schools, partners, and district.

*Stigma and Social Drivers* was a major theme with two subthemes: *Food Perceptions* and *Student Preferences & Culture*. The most coded subtheme was *Food Perceptions* which demonstrated the attitudes and impressions of the meals that may be shaped by stigma or previous experiences. For example, this quote from a student explains aspects of the meal that they dislike including taste and temperature:

“[Food Service Manager]: Any other issues, I mean really because if, I don’t know, I can’t change it.

[Student]: Just overall, it just [does not] taste good.

[District Food Service Representative]: So, think about how, we need more that would help us make an improvement. Is it too hard? Is there’s not enough flavor to it? It’s not salty enough.

[Student]: Yeah, sometimes it [does not have] too much flavor or it is just too cold.”

*Autonomy and Decision Making* has three subthemes that were all original to the inductive coding schema. The most coded subtheme was *Implementer-Driven Change*. Coded segments within this theme include ideas created by members of the school or district staff. Examples of this quote are listed in Supplement F.

*Recipient Driven Change* reflected changes and ideas driven by student or parent participants are coded to this theme. This theme was included to address the gap of recipient voices in policy implementation and demonstrate their impact on strategy development. *Student Voice* was a prominent subtheme that is defined as data representing the lack of inclusion of student voice and perspectives, efforts to prioritize their voices, and other pertinent data relating to student insights. Lastly, *Researcher Facilitation is* defined as any instance where the research team supported idea generation through giving suggestions or trying to help turn barriers into strategies. For example, any input from the researcher to strengthen the strategy implementation plan was coded under this theme (Supplement F).

*Infrastructure/Material Barriers* was the least coded parent theme, which houses two subthemes including *Policy Parameters* and *Troubleshooting/Resource Pooling*. This code is used to categorize structural or organizational challenges that impact change in operation and implementation. Several schools have dated physical structures, leading to issues with adding new equipment. Having schools with satellite kitchens leaves little room for innovating meal recipes, since the food is prepared off-site. Thus, feedback and strategy ideas that are related to *Student Preferences and Culture* are driven by lack of supportive infrastructure and focus on procurement changes. This is a common barrier that prevents diversifying meal delivery among schools in the district:

“Well, ___ talked about not having the upgrade because the building was so… The structure was so old. But what about a mobile kitchen or something like that? Because they can pull up bag and kids can actually stand in line and order. We can go up there, we can serve, but it’s actually a kitchen on wheels and they can have hot food”

*Policy Parameters* was highly prevalent due to the impact of policies and regulations on organizational operations. Constraints of USDA policy prevent additional sugar and sodium in recipes to accommodate student tastes and preferences, despite quantity and lack of flavor being a reoccurring deterrent (USDA, 2024). *Power Dynamics* was originally a singular theme that is defined as themes of empowerment within the meetings that may impact collaboration or development. Finally, *Troubleshooting/Material Barriers* was the last code added defined as challenges or logistical barriers to implementing school meals and/or challenges with meal procurement and delivery of meals and limited funding and resources for implementation.

## Discussion

Including program recipient and implementer voices in the research allowed the main stakeholders to tailor the program based on their lived experiences. Findings suggest that enhancing school meal implementation requires addressing influences related to school culture, leadership, and organizational capacity. This may include targeting local attitudes and perceptions of school meals, outreach practices to increase recipient knowledge and awareness. To our knowledge, this is the first study to include student and parent perspectives in a school-based implementation mapping process.

For Aim 1, we held collaborative planning meetings that resulted in several multi-level USM implementation strategies that can be pragmatically implemented by the programs main stakeholders. These strategies address several factors, such as the student-level stigma, lack of cohesion between schools, families, and the district, nutrition education support, cultural preferences, and communication between the schools and the district. Several of these factors were shown to be initial barriers to meal uptake in a previous needs assessment (21). Furthermore, voices that have been historically excluded from decision-making were at the forefront of policy implementation. Food service perspectives have not been prioritized in prior research regarding improvements to school meals (19). Due to their relationship with students and familiarity with school food service operations, their roles as leaders in implementation at the school should be emphasized when developing implementation strategies.

Prior research has demonstrated impactful collaboration with school staff and administration to improve physical activity programming. Several studies have highlighted the successful engagement of school staff and administration in developing implementation strategies to improve physical activity among students (15–17, 25, 32, 33). These studies are consistent with our findings from Aim 1 that show engaging a diverse group of representatives in implementation mapping strengthens strategy selection and protocol planning. Participants in our study were acutely aware of the main barriers within their community, which led to shared priorities for areas they wanted to address specifically. Furthermore, other research findings show that participant consensus drove strategy development, as they were able to select and design feasible strategies that met performance objectives (16, 17, 32). Uniquely, we opted to include students due to their frequent exposure and experiences (direct and indirect) to USM and food culture within their school settings. Students have the lived experience to understand the barriers and optimal ways to address low-student uptake of USM. Including their voices may strengthen the practicality and relevance of strategies. Byhoff and colleagues noted the lack of recipient inclusion in their studies and its potential impact on user participation rates (16). This demonstrates the significance of recipient (i.e., student) perspectives in the IM planning process.

EPIS is used widely in implementation research, but this is one of the first applications to USM implementation mapping (33–37). Previous studies have used EPIS to track and understand implementation over time (33, 34). The results of this work suggested strategies or adaptations to implementation that enhance access to their programming. Similarly, we used EPIS to track barriers and facilitators during the implementation mapping process, which guided strategy planning to ensure that the main determinants related to inner culture and organizational capacity were addressed. Although GTE is used broadly, it has yet to be included within implementation mapping. A prior study utilized GTE to categorize their methods based on their intended impact (38). Their results indicate that challenges faced can be addressed using strategies in increasing healthy options (i.e. aligned nutrition education, improving diet quality while ensuring access, etc.) (38). Similarly, our group identified several strategies that were grouped into Increase Healthy Options due to their intent to address low uptake through enhanced delivery methods.

Aim 2 findings highlighted several supports and constraints to the implementation mapping process, including supportive networks and partnerships, policy barriers to implementation, and school-driven changes based on demonstrated and perceived needs. Through inductive data analysis, our findings suggested that this work facilitated enhanced communication between the schools and the district office. There was a consistent exchange of knowledge throughout the planning meetings, enabling district staff to better understand the challenges and gaps in execution within each school. Findings from Kasprak & colleagues (39), who utilized an inductive open coding process after a series of implementation mapping meetings and grouped the results into Consolidated Framework for Implementation Research (CFIR) domains, suggest several barriers and facilitators to implementation such as structural characteristics, implementation of climate, and self-efficacy. Our findings reflect this prior work in that our inductive coding found several similar constructs influential including recipient and implementer empowerment in the implementer/recipient driven change and infrastructure and material barrier’s themes. To our knowledge, our study is novel in the use of open coding to explore themes within implementation mapping for USM enhancement.

Our mapping process relies on engagement from school community members; however, challenges to engagement in implementation mapping exist in several public health settings. Challenges we faced included timing of the meeting, implementer buy-in, and community mistrust, resulting in smaller planning groups and/or lack of consistent engagement. Other research has shown that challenges to engagement were related to partner relationships, relevance of training, lack of financial support, and overall buy-in that may have led to Lack of engagement resulted in smaller planning teams and less representative programming (40, 41). We found several similar challenges during our inductive coding process, which were assigned to constructs such as community cohesion, organizational capacity, power dynamics, and policy parameters. The themes were extracted to encompass challenges related to establishing relationships among the district, schools, and partner organizations. Planning meetings were held to facilitate these relationships and ensure changes met the needs and capacity of both implementers and recipients. While challenges were addressed during planning, they may inhibit capacity building in schools due to organizational constraints.

## Limitations

Several limitations should be considered. First, we experienced some difficulty with re-recruiting schools from our existing sample for the IM work. Several schools expressed interest in continuing the study; however, due to school capacity constraints, schools were unable to continue. Thus, we recruited two new schools without USM data from our prior needs assessment. Secondly, building trust with newer schools took time and shifted the focus of our initial conversation. A significant amount of time was spent building relationships, exploring the project’s potential, and refining ideas. Additionally, our qualitative findings may not be generalized (but methods should be widely applicable). Further, recruitment posed a challenge to mapping due to lack of flexible timing for teachers, parents, and students to join. Meetings were held during or after school hours, making this less accessible to working parents, teachers, and students. Finally, lack of consistent engagement may have hindered strategy development despite our consistent attempts to engage all participants between meetings.

## Strengths

Our study utilized a community-engaged approach that encompassed several levels of influence within and outside of the school community. Strategy development was primarily led by school community members and facilitated by the research team. Our data collection and meetings were in-person, fostering an interactive face-to-face working environment and collaboration. By including the GTE framework, we centered equity in our strategy development by identifying aspects that would benefit the most vulnerable students to food insecurity. Our data analysis followed a rigorous process, including several rounds of coding for both aims. Our Aim 1 coding scheme review was conducted with an established CAB along with several consensus meetings to establish rigor and consistency within our coding process. Aim 2 coding was completed with the support of academic and non-research peer debriefers. The debriefers offered new perspectives and themes on the data findings from the transcripts.

## Conclusion

This study introduces methods for documenting the process of implementation mapping in schools serving marginalized populations. This project addresses the need for inclusive and equitable implementation of school nutrition policies. Our findings suggest that gaps in implementation are attributable to the lack of inclusion of recipients and implementers, consideration for school culture and environment, and service operation challenges and miscommunications. Uniquely, our study has centered on the experiences of students and food service staff to implement strategies to enhance uptake of school meals pragmatically. We hope this research informs future mapping procedures that incorporate the perspectives of all impacted parties.

## Supplementary Material

Supplementary Files

This is a list of supplementary files associated with this preprint. Click to download.

- SupplementD.IMMeetingDataAnalysisProtocol.docx
- SupplementF.InductiveThematicCodingScheme.docx
- SupplementC.ImplementationMappingManual.docx
- SupplementB.SchoolImplementationCollaborationWorkbooks13.pdf

## Figures and Tables

**Table 1.** EPIS Domains and Constructs

EPIS Construct	Description
Outer Context	Influential factors that are external to the organization (i.e., school district, local & federal policies)
	Constructs:
	Service Environment Funding Leadership Inter-organizational Networks & Partnerships Responsiveness to School Community
Inner Context	Characteristics within the organization that impact implementation
	Constructs:
	Organizational Capacity Individual Adopter Characteristics Culture
Bridging Factors	Bi-directional relationships between organizations in the inner and outer context that have reciprocal influences on implementation.
Innovation Factors	Characteristics of innovation or evidence-based practice. Desired changes based on the feedback of the participants.

**Table 2.** EPIS Phases

EPIS Domain	Description
Exploration	Factors not working within the school setting. Stakeholders’ perceptions of factors that need improvement. Factors that work well within the school, community, or district setting.
Preparation	Main target areas of need and assets within the school that are based on the perception of participants. Key action steps for implementation and how leaders for strategies are identified.
Implementation	The process of testing key action steps and addressing areas of need using new strategies and/or leveraging existing strengths.
Sustainment	Plan for sustainability—the process of ensuring strategies last long-term.

**Table 3.** Getting to Equity Constructs

Construct	Facilitator	Barrier
Increase Healthy Options	Characteristics of the school environment that positively influence and/or reinforce healthy eating choices.	Characteristics of the school environment that negatively influence and/or inhibit healthy eating choices.
Reduce Deterrents	Influential strategies that disrupt barriers to healthy eating choices.	Influential barriers that interfere with healthy eating choices.
Social and Economic Resources	Programs that improve population social and economic circumstances.	Negative school community social and economic circumstances require action to reduce the burden.
Community Capacity	Enhancements within the community setting that build on existing assets.	Barriers to asset building, or a lack of existing assets and resources needed to thrive.

**Table 5.** EPIS and GTE Coding

			EPIS & GTE Themes and Constructs	
Theme	Construct	Frequency	Definition	Quote
**EPIS Phases**				
*Exploration*		98	Evaluating the setting of the evidence-based practice.	
	Barrier	46	Any mentions of what was not working in their setting, what they felt like needed improvements, and what is already working within their school cafeteria culture, administration, and student body.	I feel like there's a lot of things working against the kids in school and eating. I think one of the reasons he doesn’ want to bring a lunch is because I think the kids see it as being uncool. They don’ want to come in with their lunch, but most kids don’ do that. They have different reasons why they don’ want to eat lunch that day or why they don’ want to eat the breakfast for a couple of different reasons. So they don’ want to eat the lunch being provided. They don’ want to bring lunch. So then they just starve until they get home. -Teacher
	Facilitator	52	Assets in the outer or inner school setting that are already working and aid in effective implementation	Researcher: How many lunch options do you think there are [in total]? Food Service: Generally, if I got to think about the items tha’s actually on the menu for lunch, maybe a good 12 to 15. Parent: If the students see us eating the school lunch, can we come in one day for them to see us eating it? And if they can see us and they get our opinions and I'm like, okay. I know my son listens to me somewhat about food because he eats all my food. If he hears me say, “This was good, this is pretty good,” maybe that might change his mind a little bit to see me involved too. But school's theme song, school lunches, campaigns, all this. I just wrote it on my paper, just to make it fun, because when I was coming up school was fun. Eating lunch, that’s the time we could talk about everything that was fun.
*Preparation*		33	Strategy planning or resources, leadership, and workflow	
	Barrier	13	Lack of resources andor support to implement strategies	So taking into account that the count of students getting lunch could be high because they go through the line but some of them are just throwing that food right in the trash. And then to look at the line of students after they go through the line and to get a taste of something is wrapped around the cafeteria because I think they see that as something tha’s more tailored to the market or something that may have control over.
	Facilitator	20	Support within the inner and outer school context that expands resources for implementation	So if we could get what they want or what they like, it would satisfy that hunger and then it would really help them with academics and or social skills in the classroom - Administration
*Implementation*		1	Enacting action steps to put implementation plan into practice	
	Barrier	1	Identify key action steps for implementation and how leaders for strategies are identified.	
	Facilitator	0	who will be the champion of each strategy, what partners will provide technical assistance	
*Sustainment*		2	Embedded strategies to ensure innovation lasts long-term	
	Barrier	0	Potential challenges to long-term usage within the school environment	
	Facilitator	2	Elements within the school environment and EBP that contribute to longevity.	
**EPIS Constructs**				
*Outer Context*		*1*	Structures and processes within the inner environment	
	Service Environment - District and Vendors	55	Operations within the school district and/or meal vendors that impact implementation	The way our ordering goes, maybe with [the distributor], I think that the turnover rate for us, say if when a menu come out, we might have it like 30 days in advance but we only get the two choices. So once we lock in out the two options they give us, tha’s the options [students get]. We don’ get the option to say, oh they're not going to eat one of these choices, can we bring in this. The only option we have is the quantity that we can add. We don’ get to choose wha’s going to be actually on their menu. So I think that tha’s why I'm pushing the feedback of the item because at least if we get feedback at least we know that this menu wasn’ something [they like]. - Food Service
	Service Environment - Policies	33	Local, state, and federal sociopolitical contexts that influence implementation.	Well, and part of the issue with that is not to get on a whole other thing. So, i’s federal funding, so only certain items are reimbursable. And the reason that i’s so stringent is because God forbid taxpayer dollars go to feeding kids lunch. - School Partner
	Funding	17	Fiscal support and/or lack thereof for implementation within the school context.	You can give recommendations or say that … because a lot of times we'll go into a school and we'll say, “Well this looks like it had a kitchen back in the day, so maybe there's some structural support here.” And we'll ask the question, we'll see if there's anything that can be done. But we've been trying year after year to convert and we've kind of maximized as much as we can. Now i’s at the point we're asking the question, capital programs will have to make the decision on which school is next or if they can’ do it, or what. - District Staff
	Leadership	20	Characteristics, behaviors, and decisions of individuals with the responsibility of making decisions for the implementation strategy	was just going to say, all of you're in a position, as you're moving onto your high school, to be able to take the same concept with you. You have a voice. You will actually have gone through, obviously, the complete planning process that you can take this into the next school. If i’s not already in the school where you're headed to for high school, this is your opportunity to take it and create your own. - School Partner
	Inter-organizational Networks and Partnerships	37	Relationships and support from community partners and organizations to help strategize and build	I think i’s really, i’s going to be very site specific or what their capacity is, but I am a huge proponent for it in any way capacity that we can support that. Because I think students respecting their food or respecting food in general is something that i’s hard. You take it for granted. So you see in different areas or different areas of the United States, the way that students or children react to food. In some areas there's no waste. We, I think, a lot of times take it for granted. So I don’ know, me thinking the buy-in for seeing things grown or that you watched the whole process and seeing how you invest in something and the return of it, I don’ know. I hope that that is something that can come from this. - District Staff
	Culture	5	Characteristics of the external community and/or organziations that influence implementation	There are times where there are some rogue situations [with the food]. I'll harken back when [a distributor] had a cheesesteak, and the first day the Cheesesteak came out, the cheesesteak was on a hot dog bun with green peppers in it. We were like, “Yo, what are you doing? This is Philadelphia. You don’ do a cheesesteak like that.That is not a cheesesteak. These kids are going to revolt.” Then we went back to them and said, “Hey, you can never, ever, ever do that ever again.” So we take [feedback] to heart, we go and say, “Listen, we need to change this.” There are things that we've had completely removed because it was just bad.”
	Responsiveness to School Community	30	Leadership response to school needs and priorities in the school environment/operations.	I've been with the district for a long time. I've been in meetings like this and so I feel that food and food service and children liking food has always been a constant conversation. We always come back to the table of research and never any type of outcome because now there's still an issue with lunches and what kids want and the desire of kids. So from all of this, how is this going to get better? How is this going to build and provide quality lunches to our kids? Because again, we can do all of this and we can have a meeting about the meeting and we can have the research about the research. And at the end of the day, where does this lead us? - Teacher
Total		198		
*Inner Context*		*4*	Structures and processes within the school that impact implementation	Even with me, I'll get the lunch if I do like it or i’s appetizing to me, but tha’s not always a rare occasion. And even when I look around, not everyone always gets the lunch depending if i’s pizza, chicken nuggets, things like that. Not everyone finds the food completely appetizing because of the contents in the food. I do love the fact that there's other options and other kind of foods, but not everyone always likes that thing. Mainly pizza or meats and things like that. - Student
	Individual Adopter Characteristics	64	Unique characteristics of the individuals receiving and implementing the strategies	[They do a fruit and vegetable program tasting] at breakfast time. The leftovers will be out for lunch,if you put broccoli and cauliflower there by itself, they won’ touch it. You give them something to dip the vegetables in, they'll [eat it]. So [our partners may] give them ranch dressing, they'll eat it. - School Staff
	Culture - Inner	132	Demographics and individual characteristics of strategy adopters.	
	Organizational Capacity	73	The extent of resources, staff, time, and support within the school to implement strategies	I mean after we work out, whenever we're doing [the strategy], the sharing the meals with the staff, we would ask for volunteers and come up with a set of requirements, which I think is just easy that they would promote the school lunch program. [They] would be willing to from time to time, eat lunch with the students on a voluntary basi… we find out how many people we need and then how many people we can support and what we can do. If i’s 20 meals a week, then we can do two people a day. - Administration
Total		273		
*Bridging Factors*		*44*	Connections between outer partner organizations and inner school community to improve processes and structures for implementation	So I haven’ really been in [this school] that much this year yet, but I do come once a month for the distributions that happen. Then we just make food, maybe 50 to 75 tastings, and then give them out. We put them in the bags for students to try a sample of a healthy food with a recipe and then also an item that you can cook with. So we've done potato, peelers, spatulas, things like things that are handy in the kitchen. - SNAP-Ed Provider
*Innovation Factors*		*145*	Interactions and linkages between the school community and external organizations that influence implementation	Use mass media. Mainly I think confused computers and QR codes. Having computers on small desks like this where the windows are in the lunch room so they have a line of computers. And instead of just using that website, which we could, but we could use Google Docs, have a little voting on it and then they could just press simple button, have little emojis on it, press a button, submit it, and then repeat and repeat. Or for the QR code to have people who have phones, scan the QR code goes to Google Docs and you could just submit whatever you want. Instead of just trying to download an app, you could just use Google. Or just create a website similar to it. So tha’s one way we could use it. I think Google would just be the easiest way instead just trying to download an app and do all that. -Student
**Getting to Equity**			Characteristics of the implementation strategy	
Increase Healthy Options		178		
	Increase Healthy Options - Barrier	60	Settings and characteristics of the intervention within the setting - challenges to implementing the characteristics	The students did tell the teachers that the lunches were cold, so when he did choose to eat, they were cold. So, how would the staff members know that the lunch is hot? If you put a temperature, something on a package to make sure they know i’s hot, and it can be given to the students so the students won’ say i’s cold and then trash it. Because tha’s a waste of food. - Parent
	Increase Healthy Options - Strategy	118	Settings and characteristics of the intervention within the setting - supports to implementing the characteristics	So we do have that going on here. But then I had another one, like they were saying about have them putting out the word of the menu. They can do the menus up on the second and third floor because we already have them down here on the first, and then get involved with the menu process. When I say that, had some kids do some taste testing at certain schools because I know we tried it before. - Food Service
Reduce Deterrents		64		
	Reduce Deterrents - Barrier	29	Factors that may be a barrier or interference to the intervention	The adults turn the children away from freebies [by saying] “I don’ eat school food. How you going to eat school food? I will never eat a school food. Don’ eat that school food. - Food Service
	Reduce Deterrents - Strategy	35	Factors that alleviate barriers or interferences to the intervention	We had our staff see what it looks like in the pre-plate pack. And then we had our staff have a lunch and it was served on the line. And so it was definitely different. - District Staff
Community Capacity		60		
	Community Capacity - Barrier	12	Characteristics of the community that may limit implementation	I think [we're] being mindful of the environment and the state that we're in with our students and wha’s going on politically.We just want to make sure even in this piece and in that they have a voice but not only is their being heard, but then there's some action behind the voice. We can’ have just people coming to the table and just hearing them and just taking their time but also being able to see the impact and the effects of it - Community School Coordinator
	Community Capacity - Strategy	48	Characteristics of the community that support implementation	I think that is very powerful and we talk about it all the time. We've seen other districts do this too, where they have a day where meals are served to the entire staff so that they know wha’s on the menu, they know what is happening in the cafeterias, but it is part of regulations not compliant. - District Staff
Social and Economic		58		
	Social and Economic - Barrier	22	Social and economic circumstances that limit intervention reach	As a parent, do you guys come into the buildings and give other the service yourself? So that way i’s not [food service] doing it? Because I will tell you my child, two of them go here, they won’ eat the lunch. If they have nuggets and beans and i’s soggy, like no. So I hear you saying i’s on the website, but some parents, some families, you have to come in and give them the information because they have so many other things going on, especially when we're feeding shelter families. Their mindset is on this website. Are you guys coming in to get the services in the beginning, middle and the end because it would be helpful - Parent
	Social and Economic- Strategy	36	Social and economic circumstances that support implementation of an intervention	And then I feel like if you want to introduce something new, you have to come in maybe a week or two before and give them a preview of what i’s going to be so that way when it comes up they won’ be like, “Ew, I never…” You have to give them that taste to see what i’s going to taste like. - Dean of Students

## Data Availability

Data associated with this manuscript can be requested from the corresponding author. Supplement G. reports the information included in this qualitative report and their reference points.
